# Effects of Essential Oil Treatment on *Clostridium perfringens* Cells

**DOI:** 10.3390/pathogens15080781

**Published:** 2026-07-23

**Authors:** Safa Q. Alfattani, Mahfuzur R. Sarker

**Affiliations:** 1 Department of Microbiology, College of Science, Oregon State University, Corvallis, OR 97331, USA; safa.alfattani@oregonstate.edu; 2 Department of Biomedical Sciences, Carlson College of Veterinary Medicine, Oregon State University, Corvallis, OR 97331, USA; 3 Department of Biological Science, College of Science, University of Jeddah, Jeddah 23445, Saudi Arabia

**Keywords:** *Clostridium perfringens*, spores, essential oils, natural products, inhibition

## Abstract

*Clostridium perfringens* type F is a Gram-positive, spore-forming bacterium that causes food poisoning (FP) in humans. Our previous findings demonstrated that essential oils (EOs), including clove, rosemary, and peppermint oils, significantly inhibited the germination, outgrowth, and vegetative growth of *C. perfringens* spores. We hypothesized that cytoplasmic membrane damage could be one mechanism by which EOs exert their inhibitory effects against *C. perfringens* vegetative cells. To test this hypothesis, vegetative cells of two clinical FP strains of *C. perfringens*, SM101 and E13, were treated with EOs, and the leakage of intracellular molecules was compared between EO-treated and untreated cells. Our results demonstrated increased leakage of intracellular molecules, including ATP, 260-nm-absorbing materials, and proteins, from EO-treated cells relative to untreated cells. In addition, EO-treated cells exhibited greater crystal violet uptake than untreated cells. These results suggest that EOs induced cytoplasmic membrane damage, thereby promoting the leakage of intracellular molecules and the uptake of extracellular crystal violet. Scanning electron microscopy further confirmed cytoplasmic membrane damage by revealing substantially greater membrane disruption in EO-treated cells than in untreated cells. In conclusion, EOs may exert their anti-*C. perfringens* effects by damaging the cytoplasmic membrane, thereby disrupting membrane permeability and suppressing cell multiplication.

## 1. Introduction

*Clostridium perfringens* is a Gram-positive, anaerobic, spore-forming bacterium [1,2] that causes a wide range of diseases in humans and animals [3]. The veterinary significance of *C. perfringens* is considerable because intensive chicken production systems may serve as reservoirs for this bacterium, with implications for livestock health, including economic losses, food insecurity, and potential risks of zoonotic transmission [3]. Consequently, *C. perfringens* is considered a prevalent bacterial food contaminant [4], particularly in the retail food service sector, and has been associated with numerous foodborne outbreaks [5]. Numerous studies [6,7] have reported that *C. perfringens* produces metabolically dormant spores that are more resistant than vegetative cells to food preservation methods, including osmotic processing, high-pressure processing, moist heat, prolonged low-temperature storage, and nitrite.

Current food processing treatments used to inhibit the growth of *C. perfringens* in foods are limited by their inability to inactivate its spores [8], which can subsequently germinate into vegetative cells in food and cause disease following consumption of contaminated food [9,10]. Consequently, improved alternative approaches are needed to effectively inactivate *C. perfringens* vegetative cells, which originate primarily from spores, including natural antimicrobials and nanomaterial-based strategies for controlling *C. perfringens*-associated diseases [11]. Essential oils (EOs) are natural aromatic antibacterial agents extracted from different parts of plants, including the peel, roots, seeds, leaves, bark, flowers, and the whole plant [12,13,14]. Owing to their distinctive physicochemical characteristics and broad spectrum of biological activities, EOs have attracted increasing attention from the scientific community over the past two decades [12]. By limiting the growth of foodborne pathogens, EOs, which are generally recognized as safe, may provide an alternative approach for extending the shelf life of highly perishable food products [12]. However, because of growing interest in their use in the food processing sector and increasing consumer demand for natural and safe foods, the fundamental mechanisms underlying the antimicrobial activity of plant-derived EOs require further investigation [12]. Typically, compounds that damage bacterial membrane integrity exert rapid bactericidal or inactivating effects, and EOs and their constituents have been reported to target several cellular sites, primarily the cytoplasmic membrane [13,14]. Numerous membrane-targeting agents, such as tea tree oil [15], cause depletion of cytoplasmic components across several bacterial species.

Our previous studies demonstrated the antimicrobial activity of various natural products, including clove, rosemary, and peppermint oils, against *C. perfringens* spore germination, outgrowth, and vegetative growth in vitro [16]. In nutrient-rich trypticase–glucose–yeast extract medium, clove and peppermint oils at 0.5% (*v*/*v*) suppressed *C. perfringens* spore germination. In addition, EOs at concentrations of 0.1–0.5% blocked spore outgrowth in *C. perfringens*. Notably, treatment with 0.5% EOs completely suppressed vegetative growth of FP isolates during 6 h of incubation. However, even at a fourfold higher concentration (2%), the selected EOs did not inhibit *C. perfringens* spore formation in contaminated chicken meat under suboptimal conditions.

In this study, we hypothesized that cytoplasmic membrane damage could be one mechanism by which EOs exert their effects against *C. perfringens* vegetative cells. To investigate this hypothesis, we evaluated the effects of selected EOs, including clove, rosemary, and peppermint oils, which previously exhibited inhibitory activity against two *C. perfringens* strains. Their ability to disrupt the cytoplasmic membrane was assessed using multiple approaches, including measurements of membrane permeability, leakage of adenosine triphosphate (ATP), proteins, and nucleic acids, as well as changes in cell morphology.

## 2. Materials and Methods

### 2.1. Preparation of Bacterial Suspensions

Two *C. perfringens* type F foodborne strains were used in this study: SM101, a derivative of the type F human food poisoning (FP) strain NCTC 8798 [17], and E13, originally isolated from the feces of patients with food poisoning or from contaminated foods [18]. Stock cultures of *C. perfringens* strains were maintained at −20 °C in cooked meat medium (Difco, BD Diagnostic Systems, Sparks, MD, USA). Bacterial suspensions were prepared as follows. Aliquots (~0.1 mL) of the cooked meat stock cultures were inoculated into 10 mL of fluid thioglycolate (FTG) (BD Diagnostic Systems, Sparks, MD, USA) broth and incubated at 37 °C for 24 h. Subsequently, 0.4 mL of the overnight FTG-grown culture was transferred into 10 mL of trypticase–glucose–yeast extract (TGY) broth (3% trypticase, 2% glucose, 1% yeast extract, and 0.1% L-cysteine) and incubated at 37 °C for, 3 h. Cells from the TGY-grown cultures were harvested by centrifugation at 10,000× *g* for 15 min at 4 °C, washed twice with phosphate-buffered saline (PBS; pH 7.4), and resuspended in 5.0 mL of PBS. The optical density at 600 nm (OD_600_) was adjusted to ~3.5 and 4.5 (equivalent to ~10^6^ and 10^7^ CFU/mL) for strains SM101 and E13, respectively. Fresh bacterial suspensions were prepared for each experiment.

### 2.2. EOs and Antimicrobial Agents

Three EOs (clove, rosemary, and peppermint oils) were obtained from pharmacies and health supply stores in Corvallis, OR, USA. To prepare the desired final EO concentrations (% *v*/*v*), liquid coconut oil at room temperature was first used as a solvent before dilution in TGY broth to ensure uniform mixing and maintain fluidity. Importantly, our previous study demonstrated that coconut oil, used as a negative control, had no inhibitory effects against the tested *C. perfringens* strains [16]. The addition of EOs did not alter the pH of the TGY medium. Nisin was obtained from Sigma (St. Louis, MO, USA). A 4.0-mM nisin stock solution containing 2.5% pure nisin with an estimated efficacy of 10^6^ IU/g was prepared in 0.02 N HCl, filter sterilized (0.45 µm; Millipore, Bedford, MA, USA), and used within 1 week after storage at 4 °C.

### 2.3. ATP Leakage Assay

The percentage of ATP leakage was determined as previously described [19]. Briefly, an aliquot (1.0 mL) of freshly prepared *C. perfringens* cell suspension was inoculated into 10 mL of TGY supplemented with either 0.0016% nisin (positive control) or different concentrations of EOs (0.5%, 1%, and 2%), followed by incubation at 37 °C. After 3 and 6 h of incubation, 1 mL of each treatment was collected and centrifuged at 10,000× *g* for 10 min to separate the cell pellets from the supernatants. The cell pellets were resuspended in brain heart infusion broth. According to the manufacturer’s instructions, ATP levels in the resuspended cell pellets (intracellular) and culture supernatants (extracellular) were measured using a BacTiter-Glo kit (Promega, Madison, WI, USA). Luminescence was detected using a luminometer (Promega, Madison, WI, USA). At least two independent experiments were performed to determine the mean and standard deviation (SD) of ATP levels, which were expressed as a percentage of the untreated controls.

### 2.4. Leakage of the 260-nm-Absorbing Material

Leakage of 260-nm-absorbing material (including RNA, DNA, and proteins) was assessed according to a previously described protocol [15] with minor modifications. Briefly, an aliquot (1.0 mL) of freshly prepared *C. perfringens* cell suspension was transferred into 10 mL of PBS (pH 7.4) alone (negative control) or PBS supplemented with various concentrations of EOs, followed by incubation at 37 °C. After 3 and 6 h of incubation, 1.0-mL aliquots were collected from each control and treatment sample. The collected samples were then filtered through a 0.22-µm pore-size filter. The OD_260_ of the filtrates was subsequently measured using a SmartSpec 3000 spectrophotometer (Bio-Rad Laboratories, Hercules, CA, USA), and leakage of 260-nm-absorbing materials was calculated as follows:

OD260 of PBS/OD260 of EO-treated samples × 100.

### 2.5. Protein Leakage Assay

Protein leakage was examined using the Bradford protein assay as previously described [20] with minor modifications. Briefly, an aliquot (1.0 mL) of freshly prepared *C. perfringens* cell suspension was inoculated into 10 mL of PBS alone (negative control) or PBS supplemented with 0.0016% nisin (positive control) or different concentrations of EOs. After incubation at 37 °C for 3 and 6 h, 1.0-mL aliquots were collected from each treatment and centrifuged at 10,000× *g* for 10 min to recover the supernatants. Equal volumes of Bradford working solution and the corresponding supernatants were then mixed. Following incubation in the dark for 10 min, the OD595 was measured in triplicate. Supernatants from the appropriate dilution of each treatment served as blanks. A standard curve was generated using bovine serum albumin (provided with the Bradford protein assay kit) at known concentrations, and the concentration of leaked protein was estimated from the absorbance readings using the linear regression equation of the standard curve.

### 2.6. Crystal Violet Assay

The crystal violet assay was performed according to a previously described protocol [21] with minor modifications. Briefly, an aliquot (1.0 mL) of freshly prepared *C. perfringens* cell suspension was inoculated into 10 mL of PBS alone (negative control) or PBS supplemented with different concentrations of EOs and incubated at 37 °C for 6 h. The bacterial cells were then collected by centrifugation at 10,000× *g* for 10 min and suspended in 2.0 mL of filter-sterilized PBS containing crystal violet (10 µg/mL). After incubation in the dark at 37 °C for 15 min, the supernatants were collected, and the OD590 was measured using a SmartSpec 3000 spectrophotometer. The absorbance of the crystal violet solution was defined as 100%. Crystal violet uptake was calculated using the following formula:

(OD of the sample)/(OD of the crystal violet solution) × 100 = % uptake.

### 2.7. Scanning Electron Microscopy Analysis

Scanning electron microscopy (SEM) analysis was performed according to the method of Yong et al. (2015) [22] with slight modifications. Because all tested EOs exhibited similar inhibitory effects against *C. perfringens*, clove oil was selected to evaluate its effects on the bacterial membrane. Briefly, an aliquot (0.4 mL) of freshly prepared bacterial suspension was transferred into 10 mL of TGY alone (control) or TGY supplemented with different concentrations of clove oil. After incubation at 37 °C for 3 and 6 h, 5-mL aliquots were collected from each treatment. The suspended cells were fixed at room temperature (22 °C) in an equal volume of 5% glutaraldehyde prepared in 0.2 M sodium cacodylate buffer (pH 7.4). The samples were subsequently dehydrated through a graded ethanol series (30%, 50%, 70%, 90%, 95%, and 100%) before being critical-point dried using a Tousimis Autosamdri 931. The dried samples were then sputter-coated with gold and palladium and examined using a Quanta 3D scanning electron microscope (Thermo Fisher, Hillsboro, OR, USA). Untreated cells and cells treated with 1% and 0.5% clove oil were visualized by SEM at magnifications of 25,000× and 17,000×, respectively.

### 2.8. Statistical Analysis

All data are presented as the mean ± SD from at least two independent experiments. Mean values were compared using Prism (v10.0) (GraphPad, Boston, MA, USA). Error bars in all figures represent the SD. Statistical significance between data sets was determined using *t*-tests. A *p* value < 0.05 was considered statistically significant.

## 3. Results

### 3.1. EOs Induce ATP Leakage from C. perfringens Cells

Previous studies [19] showed that natural products (cinnamon, peppermint oil, and trans-cinnamaldehyde) significantly induce ATP leakage from *C. difficile* cells. To determine whether this effect also occurs in *C. perfringens*, we compared ATP release from *C. perfringens* cells grown in TGY supplemented with or without EOs. As a positive control, an ATP leakage assay was also performed using *C. perfringens* cultures grown in TGY supplemented with nisin.

**Figure 1:**
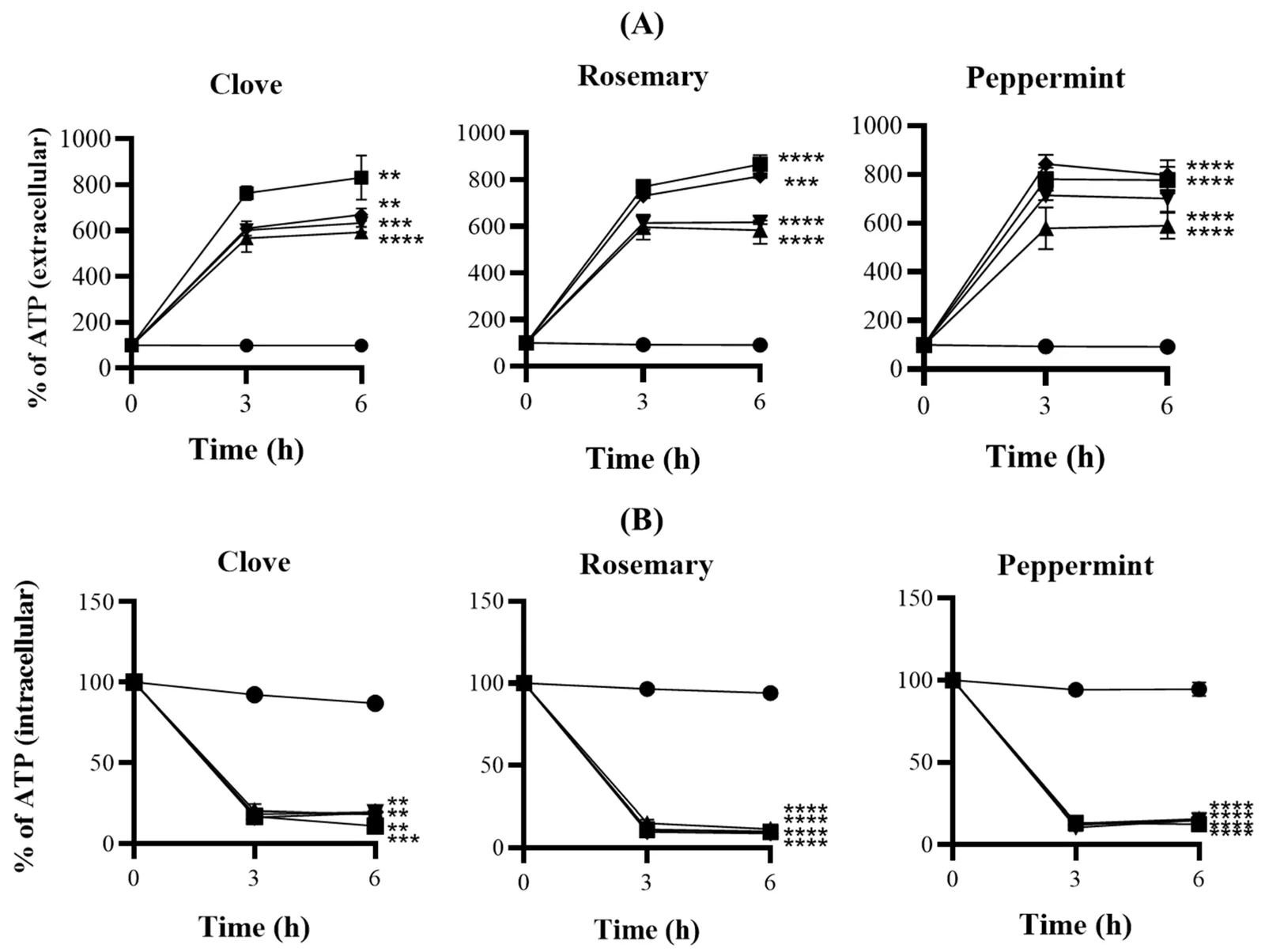
Effect of EOs on ATP leakage from *C. perfringens* strain SM101. Freshly prepared bacterial suspensions of strain SM101 were inoculated into TGY alone as a negative control (●), TGY supplemented with EOs at concentrations of 0.5% (▲), 1% (▼), or 2% (♦), or TGY supplemented with nisin (0.006%) as a positive control (■). After incubation at 37 °C, samples were collected at 3 and 6 h, and ATP levels in the supernatants (extracellular) (**A**) and resuspended cell pellets (intracellular) (**B**) were measured using the BacTiter-Glo kit. Note that, in panel B, the data represented by (▲), (▼), (♦), and (■) overlap. At least two independent experiments were performed, and ATP levels are presented as the mean ± SD and expressed as a percentage of the untreated controls. Error bars represent the SD. **: *p* < 0.01, ***: *p* < 0.001, and ****: *p* < 0.0001.

As expected, no ATP leakage was observed in *C. perfringens* strains SM101 and E13 grown in TGY alone at 37 °C for up to 6 h (Figure 1 and Figure S1). No extracellular ATP was detected in the culture supernatant, and most ATP remained intracellular. However, supplementation of TGY with nisin (0.0016%) induced a significant increase in ATP leakage from *C. perfringens* cells relative to the untreated control; a substantial amount of extracellular ATP and only a negligible amount of intracellular ATP were detected in *C. perfringens* cultures grown in TGY containing nisin (0.0016%) at 37 °C for 3 h.

Consistent with the results of nisin treatment (positive control), a significant amount of ATP was detected in the culture supernatant, whereas almost no ATP was detected in the bacterial cell pellet when *C. perfringens* was grown in TGY supplemented with various concentrations of clove, rosemary, or peppermint oil. These results indicated that all three tested EOs induced significant ATP leakage from *C. perfringens* cells, although to a lesser extent than nisin (Figure 1 and Figure S1). Collectively, these results suggest that all three tested EOs increase the permeability of *C. perfringens* cells, thereby inducing ATP leakage.

### 3.2. EOs Induce Leakage of 260-nm-Absorbing Materials from C. perfringens Cells

**Figure 2:**
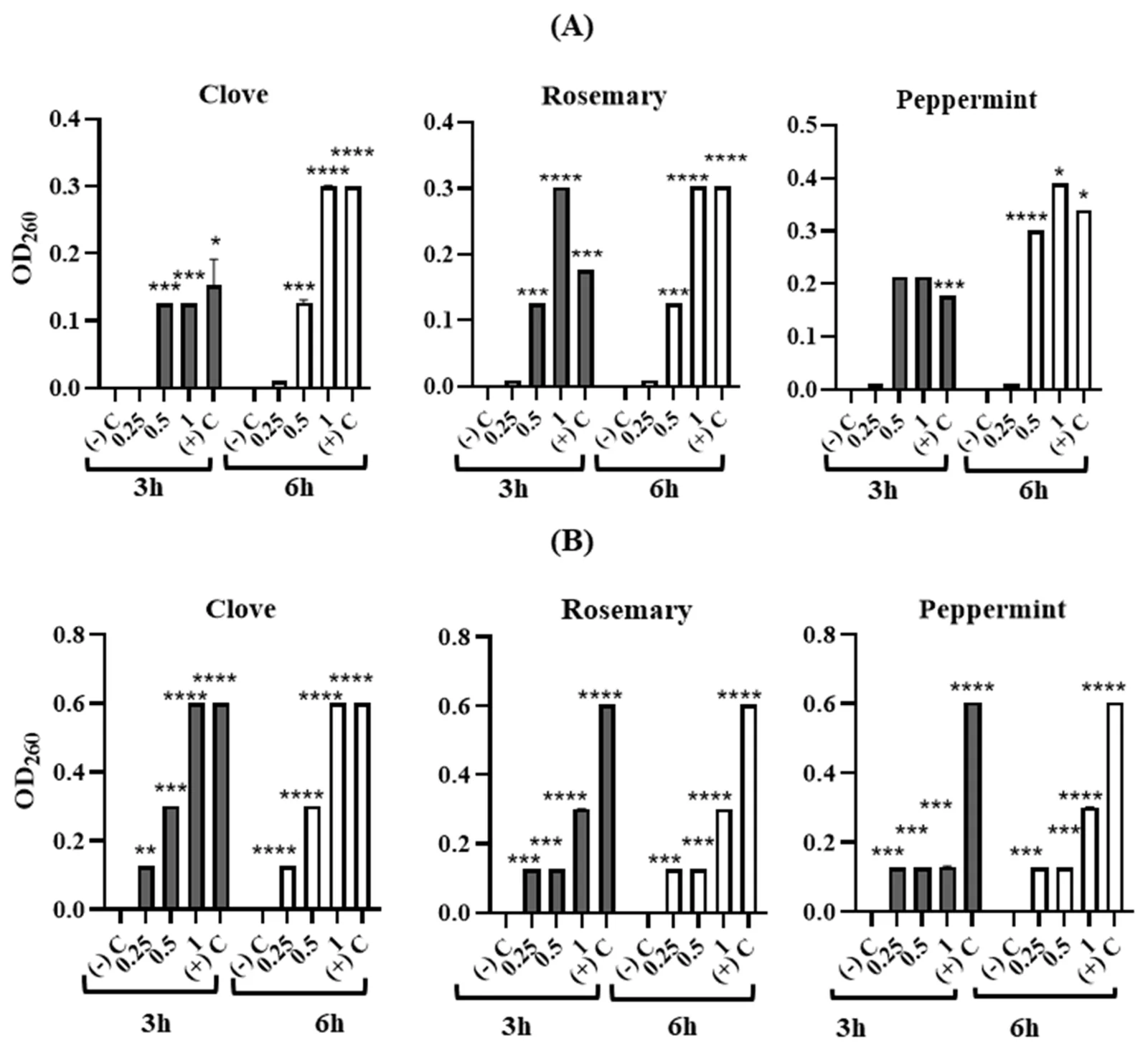
Leakage of 260-nm-absorbing materials from EO-treated *C. perfringens*. Freshly prepared cell suspensions of strains SM101 (**A**) and E13 (**B**) were inoculated into PBS alone [negative control (−C)] or PBS supplemented with the indicated concentrations (% *v*/*v*) of EOs or with 0.0016% nisin [positive control (+C)] and incubated at 37 °C. Aliquots from each treatment were collected after 3 and 6 h of incubation, and OD260 was measured as described in the Materials and Methods. At least two independent experiments were performed to determine the mean and SD. Error bars represent the SD. *: *p* < 0.05, **: *p* < 0.01, ***: *p* < 0.001, and ****: *p* < 0.0001.

Leakage of 260-nm-absorbing materials from EO-treated cells would be expected only if the EOs damaged the cell membrane. To investigate this, *C. perfringens* cells were treated with various concentrations of EOs or nisin, and the release of 260-nm-absorbing materials into the culture supernatants was monitored by measuring OD_260_. After 6 h of exposure to the antimicrobials (Figure 2), the 1% EO-treated samples and the positive control (0.0016% nisin) exhibited a significant (*p* < 0.05) increase in OD260 relative to the untreated control (PBS). After 3 and 6 h of exposure to nisin or 1% clove oil, strain E13 exhibited an approximately twofold higher OD_260_ value than strain SM101. Furthermore, significant leakage of 260-nm-absorbing material was observed at the lower EO concentration (0.25%) relative to the negative control in both *C. perfringens* strains. After 6 h of incubation at 37 °C, the positive control and 1% EO-treated samples exhibited higher levels of leaked 260-nm-absorbing material, particularly in strain SM101, suggesting that higher antimicrobial concentrations and longer exposure times were required to induce substantial leakage of intracellular contents. These findings indicate that EOs disrupt the cell membrane, thereby increasing membrane permeability and promoting the leakage of 260-nm-absorbing materials.

### 3.3. EOs Induce Protein Leakage from C. perfringens Cells

**Figure 3:**
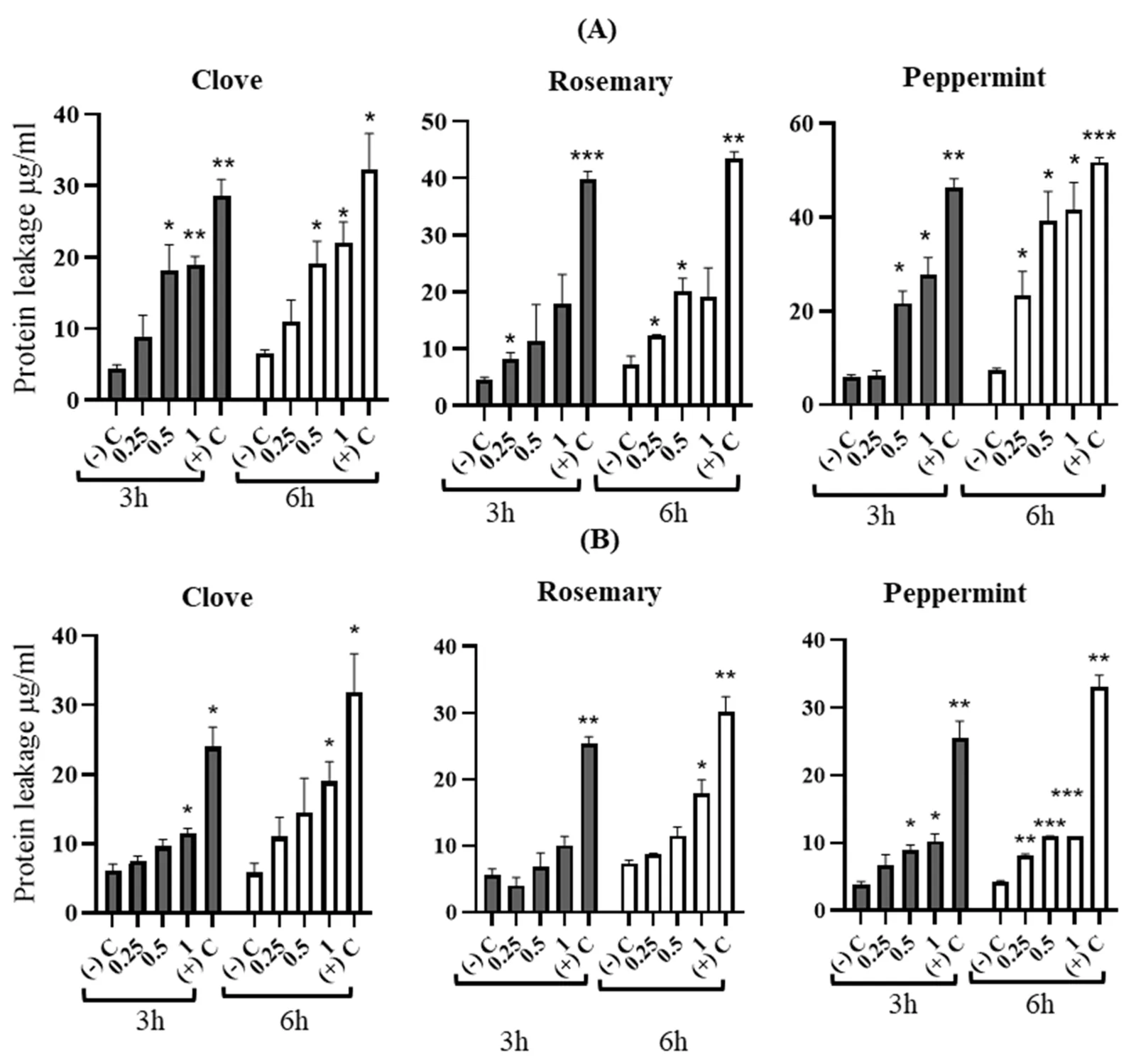
Leakage of proteins from EO-treated *C. perfringens* cells. Freshly prepared bacterial suspensions of strains SM101 (**A**) and E13 (**B**) were inoculated into PBS alone [negative control (−C)] or PBS supplemented with the indicated concentrations (% *v*/*v*) of EOs or with 0.0016% nisin [positive control (+C)] and incubated at 37 °C. Aliquots from each treatment were collected after 3 and 6 h of incubation, and protein leakage was measured as described in the Materials and Methods. At least two independent experiments were performed to determine the mean and SD. Error bars represent the SD. *: *p* < 0.05, **: *p* < 0.01, and ***: *p* < 0.001.

Next, we measured intracellular protein leakage from EO-treated *C. perfringens* cells to further confirm EO-induced cell membrane damage. *C. perfringens* cell suspensions were treated with various concentrations of EOs or nisin (positive control), and protein leakage was evaluated by monitoring increases in OD595. As expected, after 3 and 6 h of exposure to EOs or nisin, OD595 increased substantially relative to the untreated control, indicating protein leakage from *C. perfringens* cells (*p* ≤ 0.05). Although the level of protein leakage induced by EOs was lower than that induced by nisin, EO-treated cells exhibited significantly (*p* ≤ 0.05) greater protein leakage than the untreated control (Figure 3). No significant differences (*p* < 0.05) in protein leakage were observed between 3 and 6 h of incubation at any EO concentration tested. In addition, the amount of protein released from the cells increased with increasing EO concentrations (Figure 3). Interestingly, the extent of protein leakage differed between the strains, with SM101 cells exhibiting greater protein leakage than E13 cells at all EO concentrations. Collectively, these results suggest that EO treatment alters cytoplasmic membrane permeability, thereby promoting protein leakage.

### 3.4. EOs Induce Crystal Violet Absorption by C. perfringens Cells

**Figure 4:**
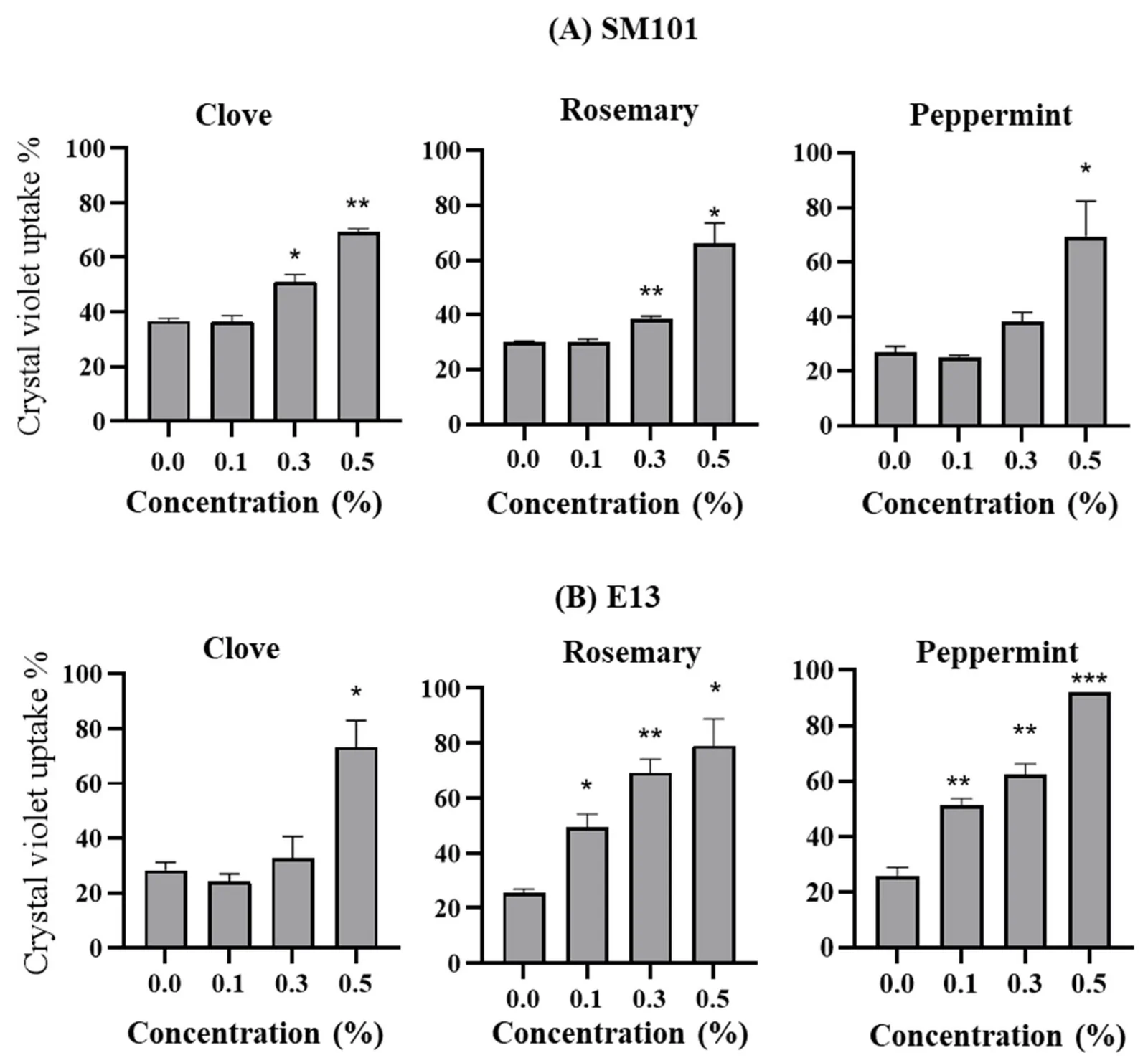
Uptake of crystal violet by EO-treated vegetative cells of *C. perfringens*. Aliquots of bacterial suspensions of strains SM101 (**A**) and E13 (**B**) were inoculated into PBS alone (negative control) or PBS supplemented with the indicated concentrations of EOs. After incubation at 37 °C for 6 h, the cells were collected and treated with 10 μg/mL crystal violet. Following centrifugation, absorbance was measured at OD590, and crystal violet uptake was calculated as described in the Materials and Methods. At least two independent experiments were performed to determine the mean and SD. Error bars represent the SD. *: *p* < 0.05, **: *p* < 0.01, and ***: *p* < 0.001.

Hydrophobic crystal violet readily penetrates damaged cell membranes but poorly permeates intact membranes. Therefore, the crystal violet uptake assay can be used to detect membrane disruption and altered membrane permeability [23,24]. Accordingly, in this study, EO-treated *C. perfringens* cells were incubated with crystal violet at 37 °C for 6 h, and crystal violet uptake was quantified by measuring OD590. As shown in Figure 4, crystal violet uptake was significantly higher in EO-treated *C. perfringens* strains SM101 and E13 than in untreated cells. Crystal violet uptake increased in proportion to the EO concentration, indicating a concentration-dependent effect. Treatment with 0.5% EOs significantly increased crystal violet uptake in both SM101 and E13 cells (*p* < 0.05, *p* < 0.01, and *p* < 0.001), reaching approximately 65–90%, compared with approximately 25–35% in untreated cells. The increased uptake of crystal violet by EO-treated cells suggests that EOs increase membrane permeability, thereby allowing crystal violet to enter the cells.

### 3.5. Clove Oil Induces Severe Damage to the C. perfringens Cell Membrane

**Figure 5:**
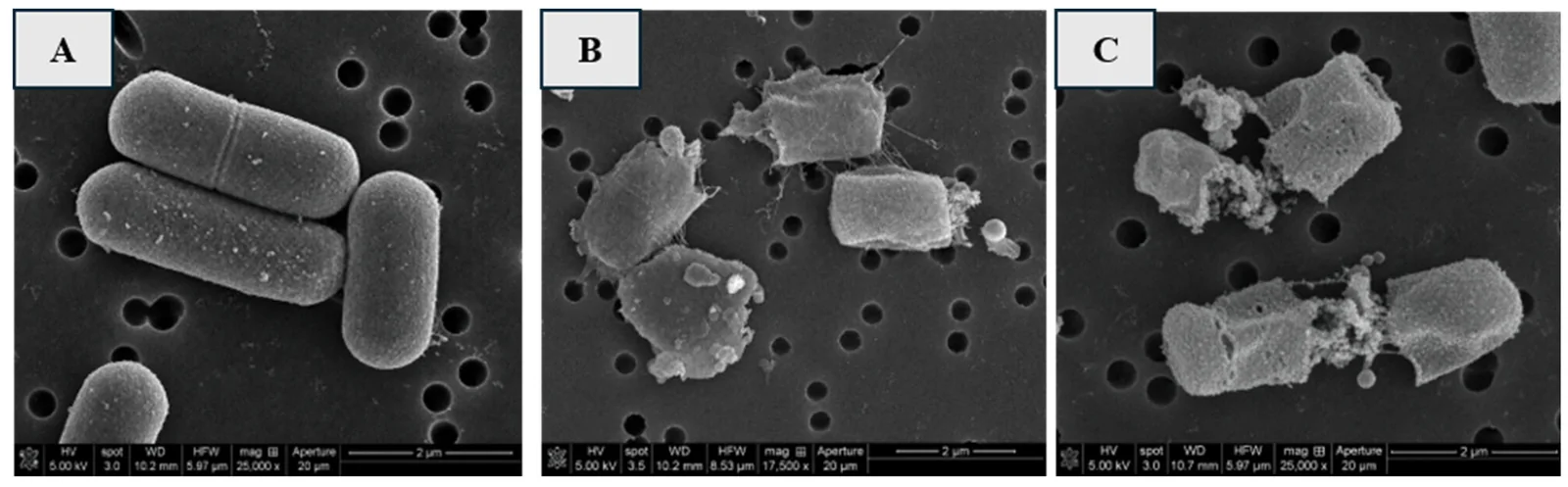
SEM of clove oil-treated vegetative cells of *C. perfringens* strain SM101. (**A**) Untreated vegetative *C. perfringens* cells. (**B**,**C**) Vegetative *C. perfringens* cells treated with 0.5% (**B**) or 1.0% (**C**) clove oil, respectively.

Having demonstrated that EOs increased membrane permeability in vegetative *C. perfringens* cells, we performed SEM to examine the morphological alterations induced by EO treatment (Figure 5). Because all three tested EOs produced similar effects on *C. perfringens* cells, SEM analysis was performed only on clove oil-treated cells.

As expected, SEM images of untreated vegetative *C. perfringens* cells showed normal cellular morphology, characterized by rod-shaped cells with smooth membrane surfaces (Figure 5A). In contrast, under the same experimental conditions, *C. perfringens* cells treated with clove oil exhibited impaired membrane integrity, damaged cell surfaces, membrane disruption, and cell lysis (Figure 5B,C). No obvious difference in the extent of cell damage was observed between cells treated with 0.5% and 1.0% clove oil, consistent with our previous findings that all evaluated EOs at 0.5% completely inhibited vegetative cell growth. However, reducing the EO concentration to 0.25% or 0.1% delayed bacterial growth relative to the control during incubation in TGY [23]. The antibacterial activity of EO constituents is generally attributed to their hydrophobic properties, which facilitate penetration of the bacterial membrane, disrupt membrane integrity, and promote the leakage of cellular components. In Gram-positive bacteria, the cell wall structure permits the translocation of hydrophobic compounds, thereby affecting both the cell wall and intracellular components [25]. Collectively, these results provide additional evidence that EOs exert their bactericidal effects against *C. perfringens* by causing severe damage to the cell membrane.

## 4. Discussion

In this follow-up study, we investigated the mechanisms of action of three EOs that previously exhibited significant inhibitory effects against different growth forms of *C. perfringens* [16]. The selected EOs markedly decreased intracellular ATP levels over 6 h of incubation and disrupted the cell membrane, as evidenced by leakage of 260-nm-absorbing materials and proteins, increased crystal violet uptake, and, finally, structural damage observed by SEM.

First, assessment of intracellular and extracellular ATP levels following EO treatment of *C. perfringens* revealed reduced intracellular ATP levels accompanied by increased ATP leakage, suggesting that all three tested EOs increased the permeability of *C. perfringens* cells, thereby promoting ATP release. Our findings are consistent with previous studies of other bacterial pathogens. For example, Roshan et al. [19] reported that intracellular ATP levels were significantly reduced (*p* ≤ 0.05) in peppermint oil-treated *C. difficile* relative to untreated controls. Furthermore, intracellular ATP levels in *Staphylococcus aureus* decreased markedly following treatment with clove oil [26]. Although the cellular mechanisms responsible for ATP depletion are complex, numerous studies suggest that exposure to membrane-active compounds promotes ATP release through membrane leakage [27].

Second, EOs induced leakage of 260-nm-absorbing materials from *C. perfringens* cells. Several previous studies have shown that such leakage occurs only when the cell membrane is damaged. Rhayour et al. [28] demonstrated that clove oil rapidly induced the release of 260-nm-absorbing materials from *E. coli* and *Bacillus subtilis*. Likewise, another study [26] showed that extracellular nucleic acids, which absorb at 260 nm, increased significantly in *S. aureus* following treatment with clove oil relative to the control group. In addition, Roshan et al. [19] reported that several natural compounds, including peppermint oil, induced the leakage of 260-nm-absorbing materials from *C. difficile*, indicating nucleic acid loss through a disrupted cell membrane. Together with these previous findings, our results indicate that EO exposure compromises the *C. perfringens* cell membrane, resulting in the leakage of macromolecules, including nucleic acids.

Next, the Bradford assay demonstrated substantial protein leakage from EO-treated *C. perfringens* cells, further indicating increased cytoplasmic membrane permeability. Consistent with previous observations in *S. aureus* [29], cell-free supernatants from EO-treated cultures contained higher protein levels than the corresponding untreated controls, confirming the membrane-permeabilizing effects of the EOs. The elevated protein levels in the cell-free supernatants suggest that EOs penetrate and disrupt the cell membrane, thereby promoting the release of soluble cellular proteins [29]. Similar findings were also reported in another study, in which exposure to natural products significantly increased protein levels in cell-free supernatants, supporting protein leakage as a consequence of EO-induced membrane permeabilization [30].

Fourth, the increased uptake of crystal violet by EO-treated *C. perfringens* cells further suggests that EOs increase membrane permeability, thereby allowing crystal violet to enter the cells. Our results are consistent with previous findings. First, crystal violet uptake by *Salmonella Typhi* increased from 43% in the absence of eugenol, the principal component of clove oil, to 95% and 99% following treatment with 1% (*v*/*v*) and 5% (*v*/*v*) eugenol, respectively [23]. Second, crystal violet uptake increased from 21.1% and 30.2% to 56.4% and 91.5% in *E. coli* and *S. aureus*, respectively, following treatment with fructose essential oil (FEO) [21].

Finally, our SEM analysis confirmed that EOs exert their bactericidal effects against *C. perfringens* by causing severe damage to the cell membrane. The loss of membrane integrity and extensive cell surface damage indicate that the tested EOs inactivate *C. perfringens* by disrupting the cell membrane and inhibiting cell proliferation. Our SEM observations are consistent with previous studies showing that clove oil and FEO alter cell morphology and disrupt the cytoplasmic membrane surface of treated cells [21,28].

This study extends previous research on selected EOs by further characterizing their antimicrobial mechanisms, particularly membrane disruption through interference with cellular components. Their application in meat products could be achieved through several delivery approaches, including direct incorporation, surface treatments, edible bio-based coatings, and active packaging. In addition, innovative strategies, such as combining EOs with bacteriophages or probiotics, may further enhance their potential for precision food safety applications.

Although EOs are known to disrupt bacterial cell membranes, as demonstrated in this study, the precise molecular targets and signaling pathways involved in *C. perfringens* remain to be identified. In addition, differences in experimental methodologies, EO sources and concentrations, exposure times, and bacterial strains make direct comparisons difficult and limit mechanistic interpretation.

## 5. Conclusions

Although the antimicrobial activities of EOs and their constituents have been extensively studied, the mechanisms of action for most EOs remain incompletely understood. Our findings demonstrate that the leakage of intracellular molecules, including ATP, 260-nm-absorbing materials, and proteins, from EO-treated *C. perfringens* cells, together with increased uptake of crystal violet, indicates EO-induced damage to the cytoplasmic membrane. This conclusion was further supported by SEM, which revealed substantial damage to the cytoplasmic membranes of EO-treated cells relative to untreated cells. In conclusion, given the membrane-permeabilizing properties of EOs, future studies should focus on evaluating their effects on the regulation of membrane proteins and identifying their molecular targets within the bacterial cell membrane. In addition, combining EOs with other food preservation strategies, such as heat treatment or other EOs, may provide an effective approach for reducing the adverse sensory effects associated with the use of high EO concentrations in food.

## Data Availability

The original contributions presented in this study are included in the article. Further inquiries can be directed to the corresponding author.

## Supplementary Materials

The following supporting information can be downloaded at: https://www.mdpi.com/article/10.3390/pathogens15080781/s1, Figure S1: Effect of EOs on ATP leakage of *C. perfringens* strain E13. Freshly prepared bacterial suspension of strain E13 was inoculated into TGY alone as a negative control (●) or TGY supplemented with EOS at a concentration of 0.5% (▲), 1% (▼), 2% (♦), or with nisin at 0.006% as a positive control (■). After incubation at 37 °C, samples were collected at 3 and 6 h, and the ATP levels in supernatants (extracellular) (A) and resuspended pellets (intracellular) (B) were assessed using a BacTiter-Glo kit. Note that, in panel B, data symbolized by (▲), (▼), (♦) and (■) overlap. At least two individual experiments were performed to determine the mean and standard deviation (SD) of ATP levels, which were expressed as a percentage of the untreated controls. The error bars represent SD from the mean,**: *p* < 0.01, ***: *p* < 0.001, and ****: *p* < 0.0001.
