# Supplementary material for: Effects of Essential Oil Treatment on Clostridium perfringens Cells
Source: Pathogens. 2026 Jul 23;15(8):781. doi: 10.3390/pathogens15080781 (PMC13516907; doi:10.3390/pathogens15080781)
Supplement: Supplementary file 1 [file pathogens-15-00781-s001.zip › pathogens-4269729-supplementary.pdf]

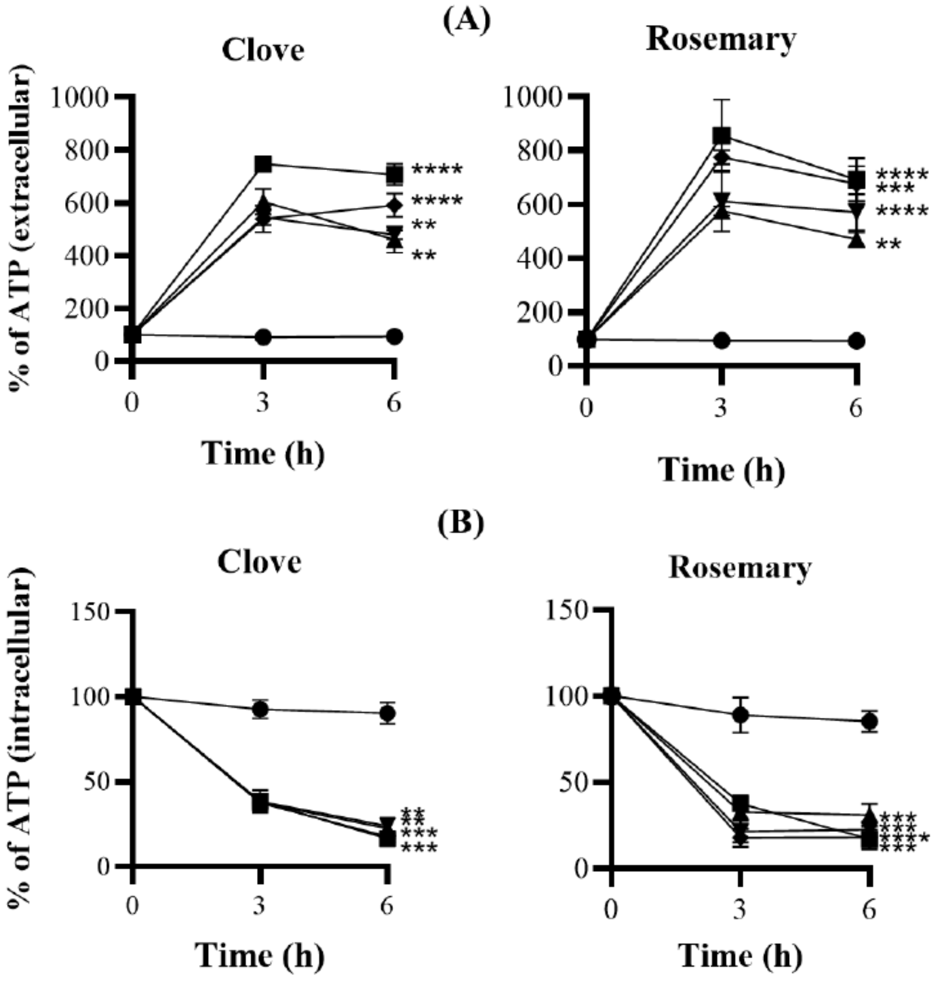

Figure S1: Effect of EOs on ATP leakage of *C. perfringens* strain E13. Freshly prepared bacterial suspension of strain E13 was inoculated into TGY alone as a negative control (●) or TGY supplemented with EOS at a concentration of 0.5% (▲), 1% (▼), 2% (♦), or with nisin at 0.006% as a positive control (■). After incubation at 37 °C, samples were collected at 3 and 6 h, and the ATP levels in supernatants (extracellular) (A) and resuspended pellets (intracellular) (B) were assessed using a BacTiter-Glo kit. Note that, in panel B, data symbolized by (▲), (▼), (♦) and (■) overlap. At least two individual experiments were performed to determine the mean and standard deviation (SD) of ATP levels, which were expressed as a percentage of the untreated controls. The error bars represent SD from the mean,**: $p < 0.01$, ***: $p < 0.001$, and ****: $p < 0.0001$.
